# Complex Regional Pain Syndrome Type II Secondary to Endovascular Aneurysm Repair

**DOI:** 10.1155/2015/954217

**Published:** 2015-01-11

**Authors:** Hamilton Chen, Sharwin Tafazoli

**Affiliations:** ^1^University of California, Irvine, Irvine, CA 92697, USA; ^2^University of California, Riverside, Riverside, CA 92521, USA

## Abstract

Complex regional pain syndrome (CRPS) is a chronic pain disorder characterized by severe pain and vasomotor and pseudomotor changes. Endovascular aneurysm repair (EVAR) of abdominal aortic aneurysms is a recent advance in vascular surgery that has allowed repair of AAA while offering reduced intensive care unit and hospital lengths of stay, reduced blood loss, fewer major complications, and more rapid recovery. Pseudoaneurysms are a rare complication of an EVAR procedure that may result in a wide range of complications. The present report examines CRPS type II as a novel consequence of pseudoaneurysm formation from brachial artery access in the EVAR procedure. To our knowledge, this is the first reported case of CRPS type II presentation as sequelae of an EVAR procedure.

## 1. Introduction

Pseudoaneurysms are a rare but existing complication of intravascular percutaneous access. Cases have been cited of pseudoaneurysm formation in the aorta, radial artery, and the brachial artery [1–6]. In the majority of these instances, the pseudoaneurysms rarely led to symptom development and when noticed were quickly operated on with no short- nor long-term effects.

An even fewer number of cases have reported minor neurological symptoms from postoperative pseudoaneurysm formation. From upper extremity polyneuropathy to brachial plexus compression, a wide array of symptoms have been reported [6, 7]. Of note, brachial artery pseudoaneurysms have produced exclusive symptoms along the distribution of the median nerve [8].

There have been very few cases of complex regional pain syndrome (CRPS) reported in the literature as a direct consequence of pseudoaneurysm formation. The only cited sources involving the eventual diagnosis of such a condition involved cardiac catheterization procedures, subsequently leading to pseudoaneurysms in the transradial and transfemoral arteries and CRPS [9, 10].

We present a novel case of CRPS type II as a complication after an endovascular aneurysm repair (EVAR) procedure for an abdominal aortic aneurysm (AAA). We discuss the initial indication, comorbidity, time lapse between symptom development and treatment, and potential methods of reducing such risks.

## 2. Case Presentation

**Figure 1:**
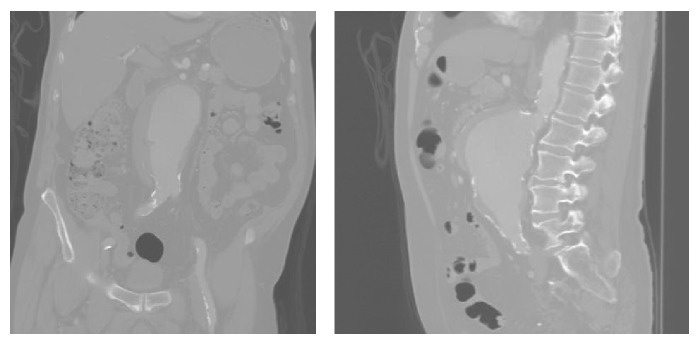
Juxtarenal AAA on CT scan.

**Figure 2:**
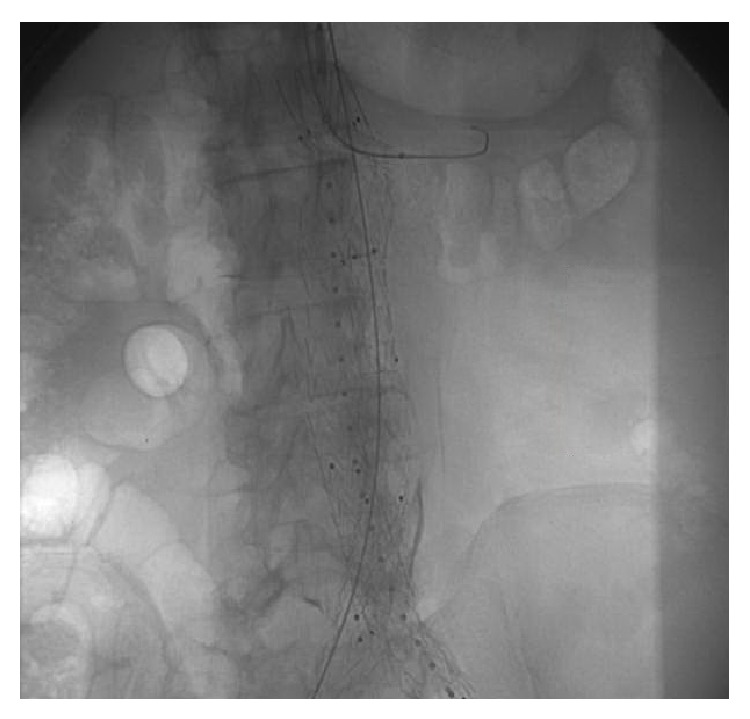
Fluoroscopy image of EVAR procedure.

A 79-year-old right-handed male with a medical history of diabetes, hypertension, coronary artery disease, and chronic kidney disease underwent the EVAR procedure with a left renal stent for an incidental finding of a juxtarenal AAA on CT scan (Figure 1). According to the vascular surgery operative report, the procedure was completed successfully through access of the bilateral femoral arteries and the left brachial artery (Figure 2). After the procedure, there was no immediate recognition of any major complications other than a small contained hematoma at the brachial catheter insertion site that formed after withdrawal of the catheter.

The patient then presented to our outpatient pain management clinic 2 months following the procedure with symptoms of atrophy and weakness of the left forearm and pain in the left hand. During the initial examination, a pulsatile mass of 2–4 cm was palpated in the medial left upper arm in the antecubital fossa. Motor assessment of the upper extremities revealed bilateral biceps flexion/extension 4/5, left finger flexion 2/5, and left hand grip 2/5. The patient's presentation was concerning for an aneurysm of the left brachial artery. An immediate ultrasound of the left upper extremity was ordered and revealed a pseudoaneurysm of the left brachial artery. The patient was urgently referred to vascular surgery and was taken to the operating room to repair the pseudoaneurysm the following day.

Four months after repair of the pseudoaneurysm, the patient returned to our clinic and his left upper extremity sequelae worsened. He now demonstrated significant guarding of his left hand and allodynia to the median nerve distribution and had skin changes and contracture of his digits with significant weakness and atrophy. An X-ray of the hand demonstrated significant osteopenia. A diagnosis of CRPS type II was established. The patient was referred for conservative treatment, which included but was not limited to pharmacologic intervention, occupational therapy for desensitization, and stellate ganglion blocks. The patient failed these conservative treatment measures, and 3 months after diagnosis he died as a result of sequelae from a respiratory infection unrelated to the current case.

## 3. Discussion

EVAR has been a major breakthrough in aneurysm repair with respect to length of hospital stay, blood loss, and complications. There is no doubt that there are significant benefits to this minimally invasive procedure. However, like any novel procedure, it poses new risks in need of serious consideration. Some of the major complications of EVAR include endoleak, migration, and structural failure.

Although not exclusive to EVAR, pseudoaneurysm formation is a possible but less common risk of endovascular procedures [1]. With the induction of a catheter through a vessel, most commonly the brachial artery, hematomas can form and both aneurysms and pseudoaneurysms can present [5, 11].

Neuropathological symptoms arising from pseudoaneurysms are a moderately common complication, depending on location of the vessel wall malformation [6, 7]. CRPS II, although notably rare sequelae, has been reported from catheter penetration through the femoral and radial arteries [9, 10]. A brachial artery pseudoaneurysm, specifically, has shown to cause median nerve paresis in a prior case report [8].

Timing between initial pseudoaneurysm detection and correction, as well as between initial development of neurological symptoms and correction, is vital in prognosis. It has been shown that if a pseudoaneurysm is diagnosed and corrected within 2 weeks of formation, the chances for complications decrease drastically [12]. When neurological symptoms have already begun, the greatest chances for complete resolution are if it is corrected within 3–5 days [13, 14].

In the case presented above, although the patient's pseudoaneurysm was corrected immediately after its discovery, this complication was not detected until well over 2 months after the initial EVAR procedure and development of neurologic sequelae. This may have contributed to the severity of his symptoms.

CRPS II is a rare complication of numerous endovascular procedures. To our knowledge, this is the first reported case of CRPS II as a complication from the EVAR procedure. The case illustrates the consequences that may ensue following a delay in diagnosis of a pseudoaneurysm following an endovascular procedure and highlights the importance of diligent follow-up after EVAR.

## References

[1] Faccenna F., Alunno A., Castiglione A., Carnevalini M., Venosi S., Gossetti B. (2013). Large aortic pseudoaneurysm and subsequent spondylodiscitis as a complication of endovascular treatment of iliac arteries. *Thoracic and Cardiovascular Surgeon*.

[2] Bhat T., Bhat H., Teli S., Rajiv B., Akhtar M., Gala B. (2013). Pseudoaneurysm a rare complication of transradial cardiac catheterization: a case report. *Vascular*.

[3] Collins N., Wainstein R., Ward M., Bhagwandeen R., Dzavik V. (2012). Pseudoaneurysm after transradial cardiac catheterization: case series and review of the literature. *Catheterization and Cardiovascular Interventions*.

[4] Pastores S. M., Marin M. L., Veith F. J., Bakal C. W., Kvetan V. (1995). Endovascular stented graft repair of a pseudoaneurysm of the subclavian artery caused by percutaneous internal jugular vein cannulation: case report. *American Journal of Critical Care*.

[5] Criado F. J., Wilson E. P., Abul-Khoudoud O., Barker C., Carpenter J., Fairman R. (2000). Brachial artery catheterization to facilitate endovascular grafting of abdominal aortic aneurysm: safety and rationale. *Journal of Vascular Surgery*.

[6] Kosmadakis G., Pappas P., Gobou A., Smirloglou D., Michail S. (2012). Severe upper extremity polyneuropathy due to inferior brachial plexus compression as a result of left subclavian artery pseudoaneurysm. *Saudi Journal of Kidney Diseases and Transplantation*.

[7] Tarng D.-C., Huang T.-P., Lin K.-P. (1998). Brachial plexus compression due to subclavian pseudoaneurysm from cannulation of jugular vein hemodialysis catheter. *American Journal of Kidney Diseases*.

[8] Pelaz Esteban M., Beltrán De Otálora S., Landeras R. M., Gallardo E., Fernández Echevarría M. A., Pérez Aguilar D. (2007). Posttraumatic pseudoaneurysm of the brachial artery and postsurgical retraction of median nerve: description of a case and ultrasonography findings. *Emergency Radiology*.

[9] Bhat T., Teli S., Bhat H. (2012). Access-site complications and their management during transradial cardiac catheterization. *Expert Review of Cardiovascular Therapy*.

[10] Saad A., Knolla R., Gupta K. (2011). Complex regional pain syndrome following transfemoral catheterization. *Journal of Invasive Cardiology*.

[11] Alvarez-Tostado J. A., Moise M. A., Bena J. F. (2009). The brachial artery: a critical access for endovascular procedures. *Journal of Vascular Surgery*.

[12] Gürel K., Gür S., Özkan U., Tekbaş G., Önder H., Oğuzkurt L. (2012). US-guided percutaneous thrombin injection of postcatheterization pseudoaneurysms. *Diagnostic and Interventional Radiology*.

[13] Roganović Z., Minić L., Savić M. (1996). Lesions of the peripheral nerves associated with pseudoaneurysms of the major arteries caused by gunshot wounds. *Vojnosanitetski Pregled*.

[14] Roganović Z., Mišović S., Kronja G., Savić M. (2007). Peripheral nerve lesions associated with missile-induced pseudoaneurysms. *Journal of Neurosurgery*.

